# Genipin Cross-Linked Decellularized Nucleus Pulposus Hydrogel-Like Cell Delivery System Induces Differentiation of ADSCs and Retards Intervertebral Disc Degeneration

**DOI:** 10.3389/fbioe.2021.807883

**Published:** 2021-12-23

**Authors:** Lei Yu, Yi Liu, Jianxin Wu, Shuang Wang, Jiangming Yu, Weiheng Wang, Xiaojian Ye

**Affiliations:** ^1^ Department of Orthopedics, Second Affiliated Hospital of Naval Medical University, Shanghai, China; ^2^ Department of Orthopedics, First Affiliated Hospital of Naval Medical University, Shanghai, China; ^3^ Department of Orthopaedics, Tongren Hospital, Shanghai Jiaotong University, Shanghai, China

**Keywords:** intervertebral disc degeneration, genipin, nucleus pulposus, ADSC, tissue engineering

## Abstract

Intervertebral disc degeneration (IDD) is the pathological basis of disc degenerative diseases (DDD). Reduction in the number of cells and degeneration of the extracellular matrix (ECM) in the nucleus pulposus (NP) are characteristics of IDD. Bio-hydrogel combined with stem cell transplantation is a promising treatment. Injectable ECM hydrogels have good biological activity and *in-situ* gelatinization. However, its biomechanics and stability are insufficient to provide adequate mechanical support for intervertebral discs and to maintain the long-term differential stimulus for seeded stem cells. In our study, we developed genipin cross-linked decellularized nucleus pulposus hydrogel (GDH) as delivery system. We evaluated the mechanical properties, stability, biocompatibility, and differentiation induction of GDH cross-linked with different concentrations of genipin *in vitro*. The GDH-loaded adipose-derived mesenchymal stem cells (ADSCs) (GDHA) were injected into the rat degenerated coccygeal intervertebral disc. The effect of intervertebral disc regeneration *in vivo* was evaluated. The results showed that GDH with 0.02% of genipin had similar elastic modulus to human nucleus pulposus, good biocompatibility, and inducibility of expressing NP-related genes. *In vivo* studies showed that GDHA improved the survival of ADSCs and improved the intervertebral height, MRI index, and histological grading score. In conclusion, GDH, as an outstanding bio-hydrogel cell delivery system, has the therapeutic potential for retarding IDD.

## Introduction

It is reported that low-back pain is currently the second leading cause of hospitalization and disability for elders, placing a serious financial burden on individuals and society (Cieza et al., 2021). Intervertebral disc degeneration (IDD) is the primary pathological basis of low-back pain. The etiology of IDD is complicated and multifaceted involving genetic, environmental, mechanical, age, and other risk factors (Henry et al., 2018). Intervertebral discs (IVDs) are composed of a central nucleus pulposus (NP), surrounded by the annulus fibrosus and cartilaginous endplates (Eyring, 1969). The NP, usually considered the origin of IDD, is the inner part and the most hydrated region (rich in proteoglycans and collagen II) of IVD transferring axial loads radially (Fontana et al., 2015). Besides the extracellular matrix (ECM) which is enzymatically degenerated during IDD, nucleus pulposus cells (NPCs) are remarkably replaced by cells of fibroblast-like phenotype (Lipson and Muir, 19801981; Lama et al., 2021). Current clinical treatments for IDD-caused low-back pain include medication intervention, surgical depression, and fusion. However, both of them cannot reverse the pathological process of IDD. Furthermore, surgical treatment accelerates the degeneration of adjacent vertebral body segments, finally leading to adjacent vertebral disease. Therefore, there is an urgent need for a satisfactory solution to reengineer the natural properties of IVD (Benneker et al., 2014).

Bioengineering of hydrogels has become a focused researching area of rebuilding the anatomy and histological structure of IVD, being deemed as a promising therapeutic method for IDD (O'Halloran and Pandit, 2007). According to the origin of composites, hydrogels are commonly classified into natural hydrogels and synthetic hydrogels. Naturally derived hydrogels are generally composed of chitosan, alginate, hyaluronan, collagen, and agarose, which can be easily acquired from various renewable bio-tissues (Mano et al., 2007; Leyva-Gómez et al., 2018). Compared with synthetic hydrogels, natural hydrogels possess inherent biocompatibility and biodegradability, while they do not have satisfactory tunability for functional facile fabrication as fine as that of synthetic hydrogels (Tang et al., 2020). Among the natural hydrogels, injectable ECM hydrogels are particularly appealing because their 3D structure and biochemical composition are highly compatible for each distinctive tissue type. ECM has been mimicking aspects of the ECM structure and composition to host tissues which have been demonstrated to dynamically and reciprocally regulate cell behavior, such as migration, proliferation, and differentiation (Geiger et al., 2001) (Cheng et al., 2014) (Young et al., 2011). In addition, ECM hydrogels can reduce immune rejecting reactions and intrinsic enzymes that allow materials to effectively integrate with surrounding tissues (Chua et al., 2016). In our previous study, we have previously developed and reported the decellularized nucleus pulposus hydrogel (DNPH), which shows good potential in NP regeneration (Yu et al., 2020). Although DNPHs have excellent biological activity, the poor mechanical properties limited their wide use in organizations with high mechanical strength requirements, such as NP (Chan et al., 2013). Moreover, current ECM hydrogels seem to not possess sufficient mechanical properties which could match the requirement of an NP biomechanical environment (Moya and Halder, 2019). Without proper mechanical properties, there would be an absence of mechanical signals from ECM bio-scaffolds such as stiffness and elasticity to stimulate host cells and transplanted cells for proliferation and differentiation (Loebel et al., 2019; Moya and Halder, 2019). Due to the mechanical properties of the IVD, stable ECM hydrogels with good mechanical properties for supporting the survival and maturation of NP are needed urgently.

A variety of mesenchymal stem cells (MSCs) have been widely used in stem cell-based IVD engineering, including cartilage or bone marrow-derived MSCs and adipose-derived MSCs (ADSCs) (Li et al., 2021). Compared with other kinds of MSC, ADSCs are especially suitable as seed cells for tissue engineering of IVD, because they are easily accessible from renewable adipose tissue and have potential to differentiate into different cells, such as NP-like cells in specific conditions (Zuk et al., 2001; Tapp et al., 2008). In addition, the prominent paracrine secretion of ADSCs is also beneficial to modulate the microenvironment of a degenerative NP environment. However, without specific exogenous stimuli, the differentiation of ADSCs is inhibited in varying degrees, especially in various hydrogels or scaffolds (Virdi and Pethe, 2021). Among those different factors affecting MSC differentiation into NP-like cells, mechanical stimuli are essential which induce MSC transformation and secretion of NP-specific ECM (Liu et al., 2021). Therefore, in the application of ADSCs in ECM-hydrogel based IVD tissue engineering, adjusting the mechanical properties of hydrogel to an appropriate degree is important for inducing ADSC differentiation and finally rebuilding the normal structure of IVD.

Genipin, a new type of natural cross-linking agent, has the advantage of having low toxicity, having a mild cross-linking process, and the cross-linking products having a good stability and biological compatibility (Ma et al., 2014; Dupont, 2016). Particularly, some studies have shown that genipin can improve the stability and mechanical strength of scaffold. Therefore, it is rational to assume that the modification of DNPH with genipin could improve its stability and biomechanical properties for better inducing the differentiation of seed cells. In this research, we developed genipin cross-linked DNPH (GDH) as a delivery system. We evaluated the mechanical properties, stability, biocompatibility, and differentiation induction of GDH with different concentrations of genipin *in vitro* to determine the proper GDH similar to the properties of NP. Then, we verified the inducing NP-like differentiation of ADSCs exerted by GDH. Finally, we injected GDH loaded with ADSCs (GDHA) into the rats’ degenerated coccygeal IVD and evaluated the rebuilding effect of GDHA on degenerated IVD *in vivo*. Our research will provide new insights and methods for exploring ECM-based injectable cell delivery systems to treat IDD.

## Materials and Methods

### Extraction, Cultivation, and Identification of ADSCs

All the animals were obtained from the Animal Center of Naval Medical University (Shanghai, China), and all procedures were approved by the Institutional Animal Care and Use Committee of the Second Military Medical University. Male SD rats of 60-g weight were anesthetized and executed to extract primary ADSCs. The sheared adipose tissue from the groin of rats was transferred into a working solution containing 0.1% collagen type I (Sigma, St. Louis, MO, USA) and 1% BSA (MP, Santa Ana, CA, USA) for digestion (37°C, 1 h). The residue and supernatant were removed by filtering and centrifuging, respectively. Then, the cells were collected and cultured. The culture medium was completely replaced once every 2 days. The third-generation ADSCs were used for subsequent experiments. The surface antigen (CD29, CD105, CD45, and CD90) positive rates of third-generation ADSCs were evaluated by flow cytometry (BD, San Jose, CA, USA) to identify the cell purity.

An induced differentiation medium (Cyagen Biosciences Inc., Santa Clara, CA, USA) was used to culture ADSCs for 3 weeks. Cell adipogenesis differentiation was verified by Oil Red O staining. Osteogenic differentiation was verified by alizarin red staining. Chondrogenic differentiation was verified by Alcian blue staining.

### Preparation of GDH and GDHA

A DNPH precursor was prepared, as reported in our previous study (Yu et al., 2020). Briefly, fresh bovine NP was subjected to a freeze-thaw cycle and 1% SDS for decellularization, followed by freeze-drying and grinding to powder. Firstly, the powder was digested with pepsin for 72 h at room temperature, then the pH and electrolyte were balanced at 4°C. Secondly, the DNPH precursor was cross-linked by genipin (with or without ADSCs) to form GDH at 37°C for 48 h. Notably, ADSCs and genipin were mixed at the same time (final cell concentration is 4 × 10^6^/ml) to form ADSC 3D culture in GDHA. Both GDH and GDHA were divided to four groups according to the concentration of genipin: 0% group (without genipin), 0.01% group, 0.02% group, and 0.04% group. The properties of the hydrogels, such as mechanical properties, stability, biocompatibility *in vitro*, and differentiation of ADSCs into NPCs, were tested.

### Detection of GDH Cross-Linking Degree

The cross-linking process was reflected by gelation kinetics (within 1 h) and ninhydrin experiments, respectively. The gelation kinetics experiment was as follows: 50-μl precooled gel precursors were placed in 96-well plates, and the absorbance was measured every 2 min for a total of 60 min at 405 nm at 37°C using a Synergy HT pacemeter (BioTek, Winooski, VT, USA). The data were normalized according to Eq. 1. The ninhydrin experiment was follows: growth hormone deficiency (GHD) with different cross-linking times was added to a ninhydrin solution (solution of ethylene glycol monomethyl ether with 8.5/ml mg of ninhydrin and 1.5 ml/mg of hydrindantin) and incubated in boiling water for 15 min, followed by cooling to room temperature and diluting with 60% ethanol. The absorbance of the solution at 570 nm was recorded with the Synergy HT pacemeter (BioTek, Winooski, VT, USA). The amount of free amino is proportional to the absorbance of the solution. The cross-linking degree is calculated as shown in Eq. 2.

normalized absorbance =A-A0/Amax A0 (1)

A is the absorbance, A0 is the initial absorbance, and Amax is the maximum absorbance.

cross-linking degree = 1−OD(cross-linking)/OD(uncross-linking)×100% (2)

### Mechanical Determination of GDH

The GDH in each group was trimmed into cylinders with a diameter of 0.7 cm and height of 0.5 cm. An Instron 4455 universal material testing machine (Biomedical Center, Donghua University, Shanghai, China) was used to perform mechanical testing. The maximum stress *σ* (kPa) and maximum strain (%) of each sample were measured according to the instrument specification, and the elastic modulus E (kPa) of the samples was calculated. The calculation formula is E = *σ*/ε.

### Biodegradation of GDH *in vitro*

The type I collagenase solution (50 U/ml) with double antibodies (Thermo Fisher Scientific, Waltham, MA, USA) was used as the degradation solution, and the ratio of hydrogel volume (ml): degradation solution (ml) is 1:20 (Butterfield et al., 2011). The mixture was subjected to vibration and at constant temperature of 37°C. The total time of complete degradation was recorded.

### Microstructure

GDH in each group was frozen quickly with liquid nitrogen and cross-cut by a blade. The hydrogel was observed *via* cryogenic scanning electron microscopy (SEM, FEI, USA). The average mesh area was measured and calculated by software of ImageJ.

### Biocompatible Assay

GDHAs, also divided into 0%, 0.01%, 0.02%, and 0.04% groups, were prepared for three-dimensional culture. GDHAs (200 μl) were seeded in 12-well plates, and the complete medium was changed every 2 days. The monolayer culture was used as a control group. Cell proliferation and cytotoxicity were detected respectively by CCK-8 staining (Dojindo, Kumamoto, Japan) at 1, 3, and 7 days and live/dead staining at 5 days (R37601 Life Technologies, Carlsbad, CA, USA) according to the manufacturer’s protocol. The number of living cells was measured by software of ImageJ, and the living cell ratio was calculated to reflect the cell viability.

### NP Phenotype Expression of ADSCs Encapsulated in GDHA

We chose 0% and 0.02% groups of GDH for ADSC 3D culture, and the monolayer culture was used as a control group. The expression levels of NP phenotype genes (collagen I, collagen II, aggrecan, sox-9) were detected at 7 and 14 days. The primers used for RT-PCR are shown in Table 1. The procedures were carried out as described in our previous report. Briefly, total RNA was extracted using TRIzol reagent (Thermo Fisher Scientific). cDNA was synthesized by reverse transcription, followed by real-time PCR utilizing the SYBR Premix qPCR Kit (Takara, Shiga, Japan). GAPDH was used for normalization. Relative gene expression data were conducted by the 2-ΔΔCT method. Each sample was tested in triplicate.

**TABLE 1:** Primers used for RT-PCR analysis.

Gene	Forward primer (5′–3′)	Reverse primer (5′–3′)
Collagen I	GCT​CCT​CTT​AGG​GGC​CAC​T	ATT​GGG​GAC​CCT​TAG​GCC​AT
Collagen II	GGG​TCA​CAG​AGG​TTA​CCC​AG	ACC​AGG​GGA​ACC​ACT​CTC​AC
Aggrecan	AGG​TGT​CGC​TCC​CCA​ACT​AT	CTT​CAC​AGC​GGT​AGA​TCC​CAG
sox-9	AGT​ACC​CGC​ATC​TGC​ACA​AC	ACG​AAG​GGT​CTC​TTC​TCG​CT
GADPH	AGG​TCG​GTG​TGA​ACG​GAT​TTG	GGG​GTC​GTT​GAT​GGC​AAC​A

After being 2D cultured on the surface of 0% or 0.2% GDH, ADSCs were scraped off and lysed by RIPA buffer. After being quantified with a BCA protein assay kit (Thermo Fisher Scientific Inc., Waltham, MA), the proteins were separated by SDS-PAGE and transferred onto polyvinylidene fluoride (PVDF) membranes. After being blocked with 5% bovine serum albumin (BSA), the PVDF membranes were incubated with primary antibodies: collagen I (ab260043, Abcam, Cambridge, MA, USA) (ab205718); collagen II (GB11021, Servicebio, Wuhan, China); aggrecan (sc166951, Santa Cruz, Dallas, TX, USA); sox9 (ab185966, Abcam, USA); and GAPDH (ab8245). Then, the membranes were washed and incubated with species-matched peroxidase-conjugated secondary antibodies (Beyotime, Shanghai, China) at room temperature for 2 h, and an ECL kit (Millipore, Bedford, MA, USA) was used to visualize the immunoreactive bands. Finally, ImageJ software was used to calculate the band density.

### Animal and Surgical Procedures

Eighty male SD rats 3 months old were used in this experiment. When the rats hit 3 months, skeletal maturity was reached and the IVD remodeling proved irrelevant to animal growth (Hughes and Tanner, 1970). The disc degenerative disease (DDD) model was established as described by Rousseau et al. (2007). After rat anesthesia, the vertebral bodies of Co7-9 were found by palpation and confirmed by X-ray. Needles (20 G) were inserted (parallel to the end plates) into the center of the NP, rotated to 360°, and held for 30 s. After 2 weeks, 2 μl of injection in each group was injected using a microsyringe attached to a 31-G needle into the disc, and the rats were kept still (for gel *in situ*) for more than 30 min. We defined the injection time as the starting time (0 weeks). These rats were divided into 5 groups randomly: sham group (without needle puncture and treatment), control group (needle puncture and DMEM injection, ADSC group (needle puncture and ADSC injection), GDH group (needle puncture and 0.02% GDH injection), and GDHA (needle puncture and 0.02% GDHA injection). Then, the degeneration of the rat tail IVD was evaluated at 0, 4, 8, and 12 weeks after injection.

### X-Ray and MRI

At the time of 0, 4, 8, and 12 weeks after injection, the rats were anesthesia by pentobarbital sodium (50 mg/kg, intraperitoneal injection) and placed on the platform in prone position. The radiographic beam (GE Medical Systems, Buckinghamshire, UK) was adjusted to focus at the 2 target discs. Radiographs and disc height index (DHI) were obtained using the image unit with a collimator-to-film distance of 66 cm, exposure of 63 mA, and penetration power of 35 kV. At 0 and 12 weeks after injection, T2 weighting with a 7.0-T MRI scanner (GE Medical Systems, UK) was used to reveal the water content and the structure of the IVD. The parameter settings were as follows: spin echo repetition time, 2,275 ms; echo time, 80 ms; number of excitations, 8; field of view, 5 cm; slice thickness, 1.5 mm; and no phase wrap. The MRI index (NP area × T2 signal intensity) was measured by Sante DICOM free software.

### H and E and Safranin O Fast Green Stain

At the time of 0, 4, 8, and 12 weeks after injection, the rats were executed. The harvested samples were fixed by 4% paraformaldehyde, followed by de-calcification and paraffin-embedded sectioning (5 μm). The standard sagittal position is maintained during section. H and E stain and Safranin O-fast green stain were performed respectively. The histologic scores were evaluated according to the cellularity and morphology of the IVD (Table 2) (Han et al., 2008).

**TABLE 2:** Histological grading scale.

Cellularity and morphology	Grade
Cellularity of the AF	1. Fibroblasts comprise more than 75% of the cells
	2. Neither fibroblasts nor chondrocytes comprise more than 75% of the cells
	3. Chondrocytes comprise more than 75% of the cells
Morphology of the AF	1. Well-organized collagen lamellae without ruptured or serpentine fibers
	2. Inward bulging, ruptured, or serpentine fibers in less than one-third of the annulus
	3. Inward bulging, ruptured, or serpentine fibers in more than one-third of the annulus
Border between the AF and NP	1. Normal, without any interruption
	2. Minimal interruption
	3. Moderate or severe interruption
Cellularity of the NP	1. Normal cellularity with stellar-shaped nuclear cells evenly distributed throughout the nucleus
	2. Slight decrease in the number of cells with some clustering
	3. Moderate or severe decrease (>50%) in the number of cells with all the remaining cells clustered and separated by dense areas of proteoglycans
Morphology of the NP	1. Round, comprising at least half of the disc area in mid-sagittal sections
	2. Rounded or irregularly shaped, comprising one quarter to half of the disc area in mid-sagittal sections
	3. Irregularly shaped, comprising less than one quarter of the disc area in mid-sagittal section

### Evaluation of the Survival and Migration of the Transplanted ADSCs

To track the ADSCs in degenerative discs, GFP-ADSCs were used in the ADSC group and the GDHA group. GFP-ADSCs were obtained from 10 green fluorescent protein (GFP)-transgenic female SD rats [50–60 g, SD-Tg (CAG-enhanced GFP) CZ-004Osb, Sina-British SIPPR/BK Lab, Animal Ltd., Shanghai, China]. The animal model established was as mentioned above. The samples were collected and cross-sectioned (5 μm) with a freezing microtome (Leica, Wetzlar, Germany) at 0, 4, 8, and 12 weeks, respectively. Then, the specimens were stained with DAPI (Servicebio, Wuhan, China) for 3 min. The survival of GFP-ADSCs excited green fluorescence under a fluorescence microscope (Leica, Wetzlar, Germany).

### Statistical Analysis

Continuous variables are presented as the mean ± SD, while categorical variables are presented as quartiles. Student’s t test was used for two-group comparison, while one-way ANOVA test was used for multiple groups and intergroup comparison was assessed by the LSD t-test. *p* < 0.05 was considered statistically significant. All data were analyzed using SPSS 20.0 (IBM, Armonk, NY, USA) software. GraphPad Prism 6.0 was used for data conversion and generating the statistical graph.

## Results

### Characterization of ADSCs

The results of the flow cytometry detection of cell surface antigen of ADSCs showed that the expressions of CD29 and CD90 were positive (positive rate >90%), while the expressions of CD44 and CD45 were negative (positive rate <5%, Supplementary Figures S1A–C). The ADSCs were cultured under different induced differentiation conditions and stained accordingly. After 2 weeks of adipogenic induction, a large number of red-stained fat droplets were observed in the cytoplasm after oil Red O staining. After 2 weeks of osteogenic induction, alizarin red staining showed red calcium nodules. After chondrogenic induction for about 3 weeks, Alcian blue staining showed the blue matrix inside and outside the cells (Supplementary Figures S1D–G). Both flow cytometry and differentiation experiments proved that most of the obtained cells were stem cells with good differentiation potential, which can be used for further experiments.

### Observation and Cross-Linking Degree Detection of GDH

**FIGURE 1:**
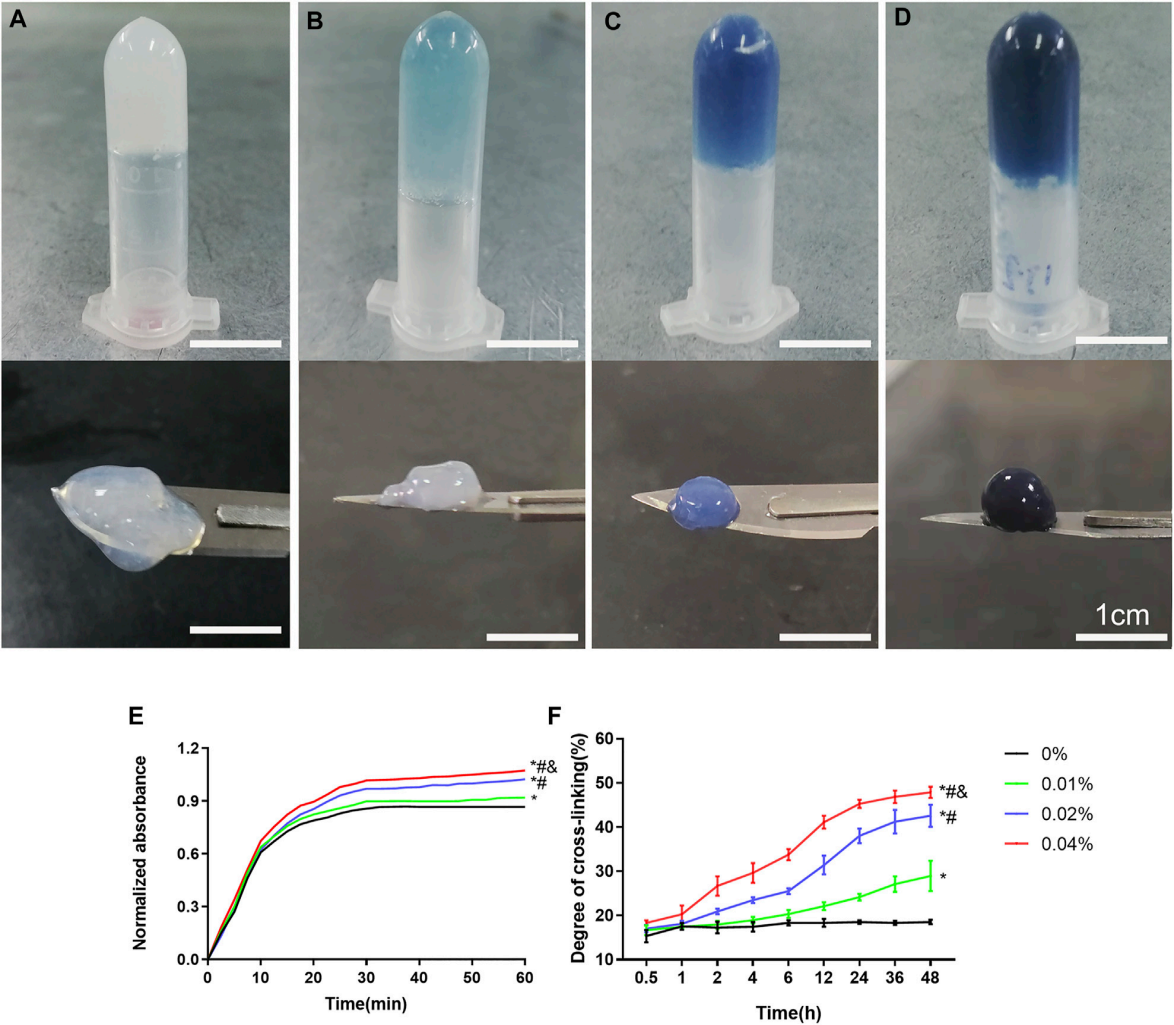
The appearance and cross-linking degree of the GDH cross-linked by different concentrations of genipin for 48 h. The morphology and color of GDH shown in the 0% **(A)**, 0.01% **(B)**, 0.02% **(C),** and 0.04% **(D)** groups. **(E)**: Gelation kinetics curve; **(F)**: time-variant degree of cross-linking measured by the ninhydrin experiment. Data represented mean ± SD (*n* = 5); *p < 0.05, vs. 0% group; ^#^ *p* < 0.05, vs. 0.1% group; ^&^ *p* < 0.05, vs. 0.02% group. Scale bar = 1 cm.

The precursor of DNPH was mixed with different concentrations of genipin evenly and placed at 37°C. After 30 min, all groups were presented as opalescent translucent gel (GDH). Over time, GDH became blue and deepens gradually. After 48 h, the color of GDH was basically no longer deepened. At 48 h, the 0% group is a milky white and semi-fluid gel with a low cross-linking degree (Figure 1A). In the 0.01% group, the appearance was light blue and the cross-linking degree increased (Figure 1B); the 0.02% group with blue appearance has a higher cross-linking degree (Figure 1C). The 0.04% group has a dark blue appearance with the highest cross-linking degree (Figure 1D).

The gelation kinetics curve was obtained, and the trend of curve in each group was roughly similar (Figure 1E). The 0% group completed the cross-linking in about 30 min, while other groups were still cross-linking slowly after 30 min. The time-variant degree of cross-linking was measured for 48 h (once an hour) by the ninhydrin experiment (Figure 1F). The 0% group was basically stable within 1 h, while other groups showed a slow rise until 48 h. At the same time point, the cross-linking degree of GDH increased significantly with the increase in the concentration of genipin (*p* < 0.05%).

### Microstructure

**FIGURE 2:**
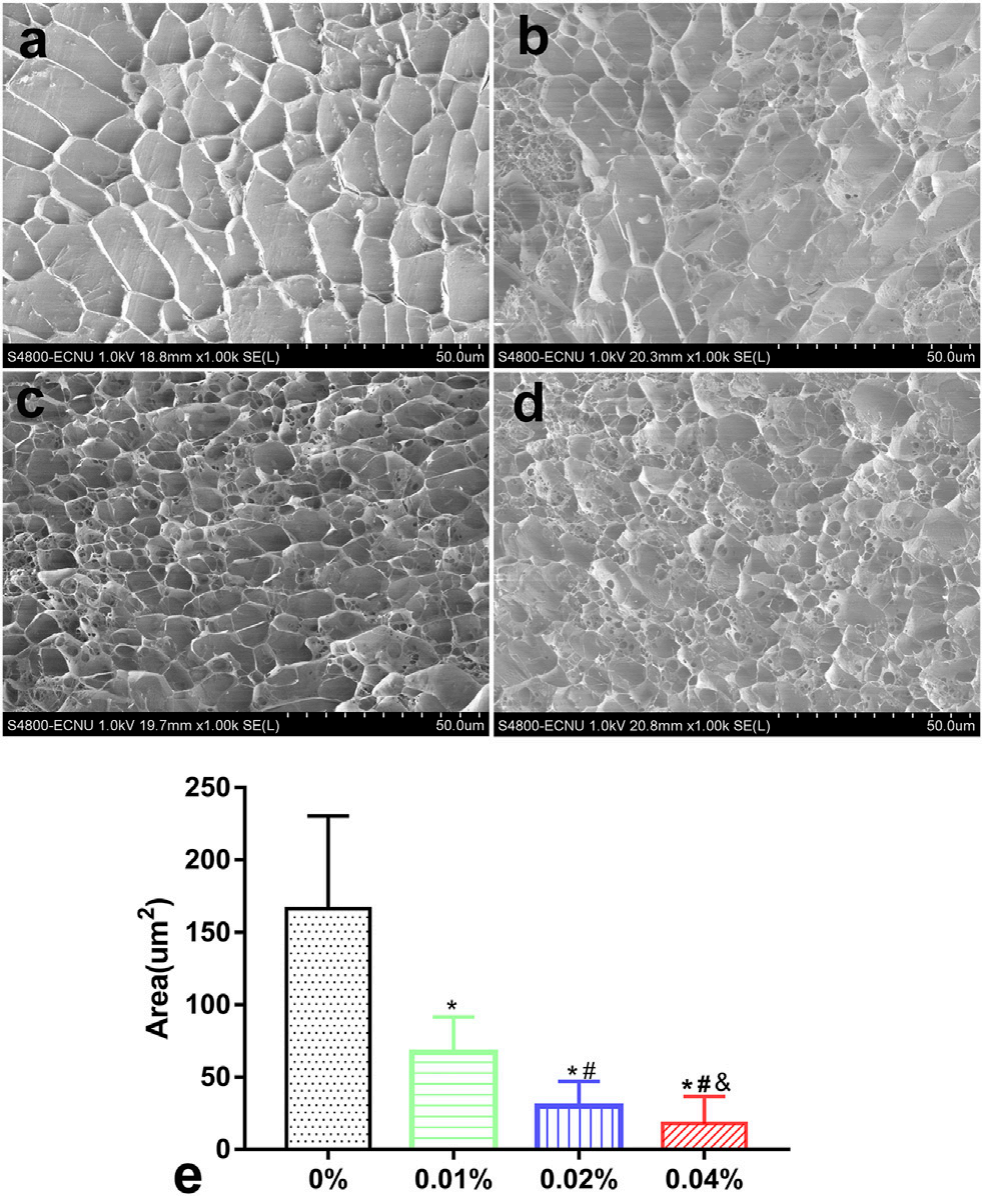
The GHD microstructure observation and grid area measurement by a cryoelectron microscope. Cryoelectron microscope images of GHD in the **(A)** 0%, **(B)** 0.01%, **(C)** 0.02%, and **(D)** 0.04% groups. **(E)** The quantification of the average grid area of GHD cross-linked with genipin. Data represented mean ± SD (*n* = 5); *p < 0.05, vs. 0% group; ^#^ *p* < 0.05, vs. 0.1% group; ^&^ *p* < 0.05, vs. 0.02% group.

Hydrogels in each group appeared as a grid under a freezing microscope. The microstructure of the 0% group was porous with a smooth and regular skeleton (Figure 2A). With the addition of genipin, the microstructure of GDH became twisted and tough, and the pores became more irregular. GDH formed a new cross-linking on the basis of the original grid structure of DNPH. As the concentration of genipin increased, the microstructural transformation of the hydrogel became more and more obvious (Figures 2B–D). The average grid area, measured by software ImageJ, decreased significantly with the increase in genipin concentration (*p* < 0.05%, Figure 2E).

### Mechanical and Stability Test

**FIGURE 3:**
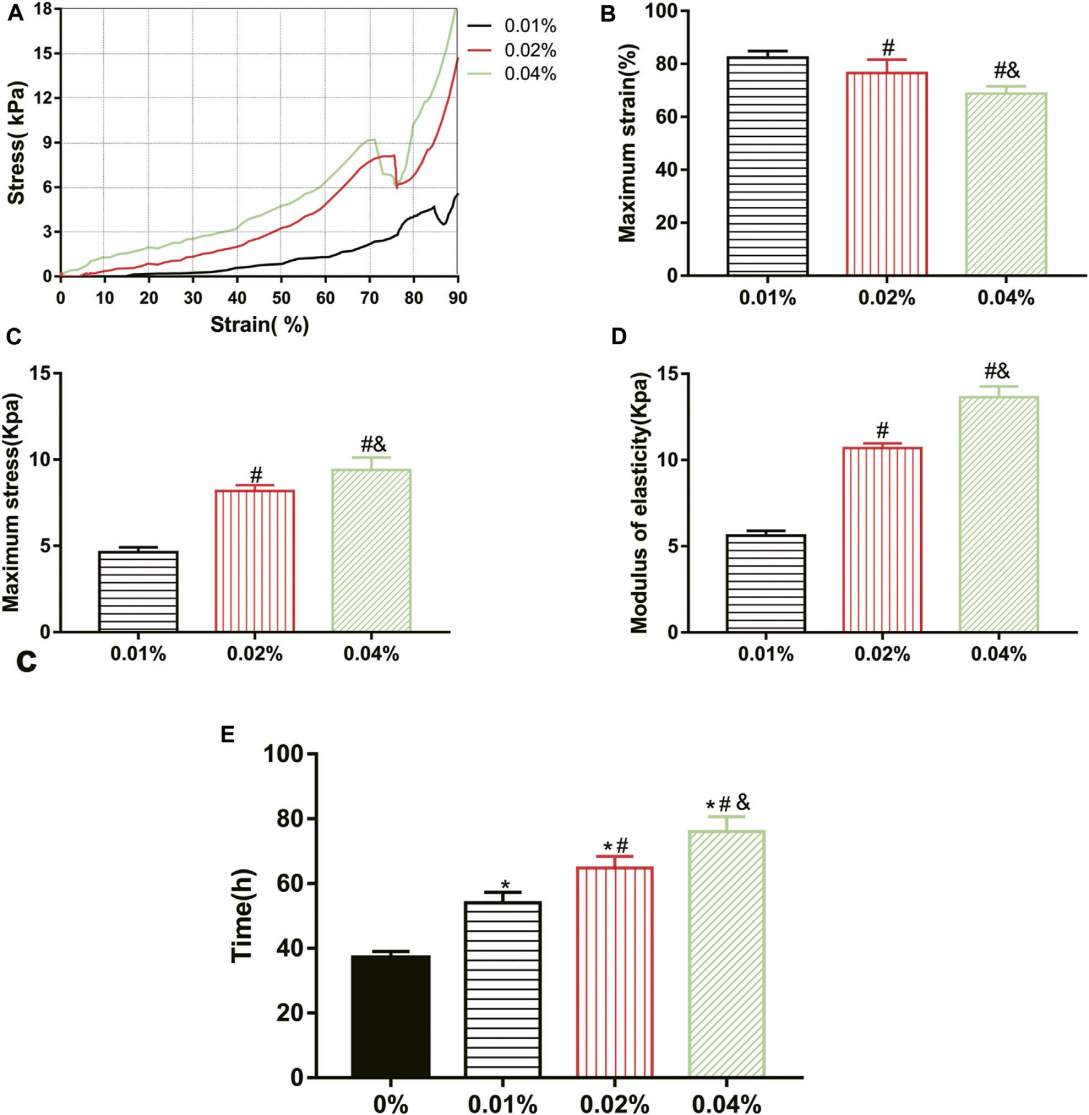
Effect of genipin concentration on GDH cross-linking rule and stability. **(A)** The stress–strain curves of the GDH hydrogels with different genipin concentrations; **(B,C)** the maximum strain and maximum stress of the GDH hydrogels with different genipin concentrations; **(D)** the elastic modulus of the GDH hydrogels with different genipin concentrations; **(E)** the stability of GDH reflected by the time of complete degradation *in vitro*. Data represented mean ± SD (*n* = 5); *p < 0.05, vs. 0% group; ^#^ *p* < 0.05, vs. 0.1% group; ^&^ *p* < 0.05, vs. 0.02% group.

All groups completed the mechanical test except the 0% group, which was a too loose structure to test. The maximum stress, maximum strain, and elastic modulus of GDH at the inflection point (when the hydrogel was destroyed) were obtained according to the corresponding stress–strain curve (Figure 3A). Results showed that the maximum strain of the 0.01%, 0.02%, and 0.04% groups was (82.36 ± 2.50)%, (76.57 ± 5.09)%, and (68.82 ± 2.73)%, respectively; the maximum stress of each group was (4.63 ± 0.29) kPa, (8.18 ± 0.33) kPa, and (9.40 ± 0.73) kPa; and the elastic moduli were (5.62 ± 0.28) kPa, (10.70 ± 0.28) kPa, and (13.64 ± 0.63) kPa. Therefore, with the increase in genipin concentration, the maximum strain of GDH decreased significantly (*p* < 0.05, Figure 3B), and the maximum stress and elastic modulus increased significantly (*p* < 0.05, Figures 3C,D). This suggested that the elastic modulus of the corresponding GDH increased with the increase in the concentration of genipin.

The biodegradation of GDH *in vitro* was also tested. The volume of each gel gradually decreased due to the action of collagenase, and the complete degradation time of each group is shown in Figure 3E. The stability of GDH increased significantly with the increase in cross-linking degree (*p* < 0.05%).

### Biocompatibility *in vitro*

**FIGURE 4:**
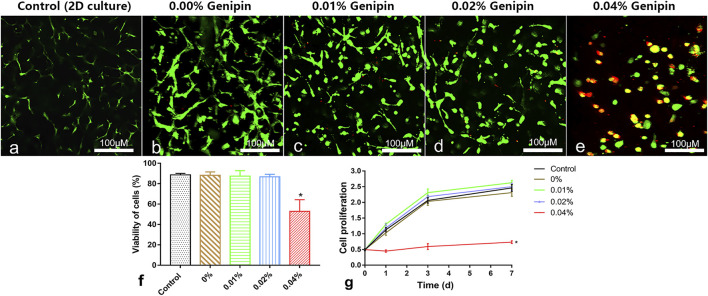
Cytocompatibility measurement of the hydrogels *in vitro*. **(A–E)** Live/dead cell staining and cell morphology observed at 5 days in the 2D-cultured control group and 0%, 0.01%, 0.02%, and 0.04% groups; **(F)** the living cell ratio showing the viability of cells; **(G)** the cell proliferation tested by the CCK-8 test. Data represented mean ± SD (*n* = 5); *p < 0.05, vs. 0% group; ^#^ *p* < 0.05, vs. 0.1% group; ^&^ *p* < 0.05, vs. 0.02% group. Scale bar = 100 μm.

Live/dead staining showed that the vast majority of cells in the control group (2D monolayer culture) and the 0%, 0.01%, and 0.02% groups were green stained (live cells); few cells were red (death cells, Figures 4A–E). After counting the live/dead cells, the living cell ratios were 91%, 90%, 87%, and 88%, respectively, with no significant difference between groups (control, 0%, 0.01%, 0.02% group), while a large number of red-stained cells were observed in the 0.04% group, and the living cell ratio was 52%, which was significantly lower than that in the other groups (*p* < 0.05%, Figures 4F). The CCK-8 test showed that there were no significant differences of cell proliferation between the control and 0%, 0.01%, and 0.02% groups (*p* > 0.05). However, the cell proliferation in the 0.04% group was much lower than that in other groups (*p* < 0.05, Figures 4G). Giving a comprehensive consideration to the stability and biocompatibility of GDH, we determined that the proper concentration of genipin for cross-linking was 0.02%.

### NP-like Differentiation

**FIGURE 5:**
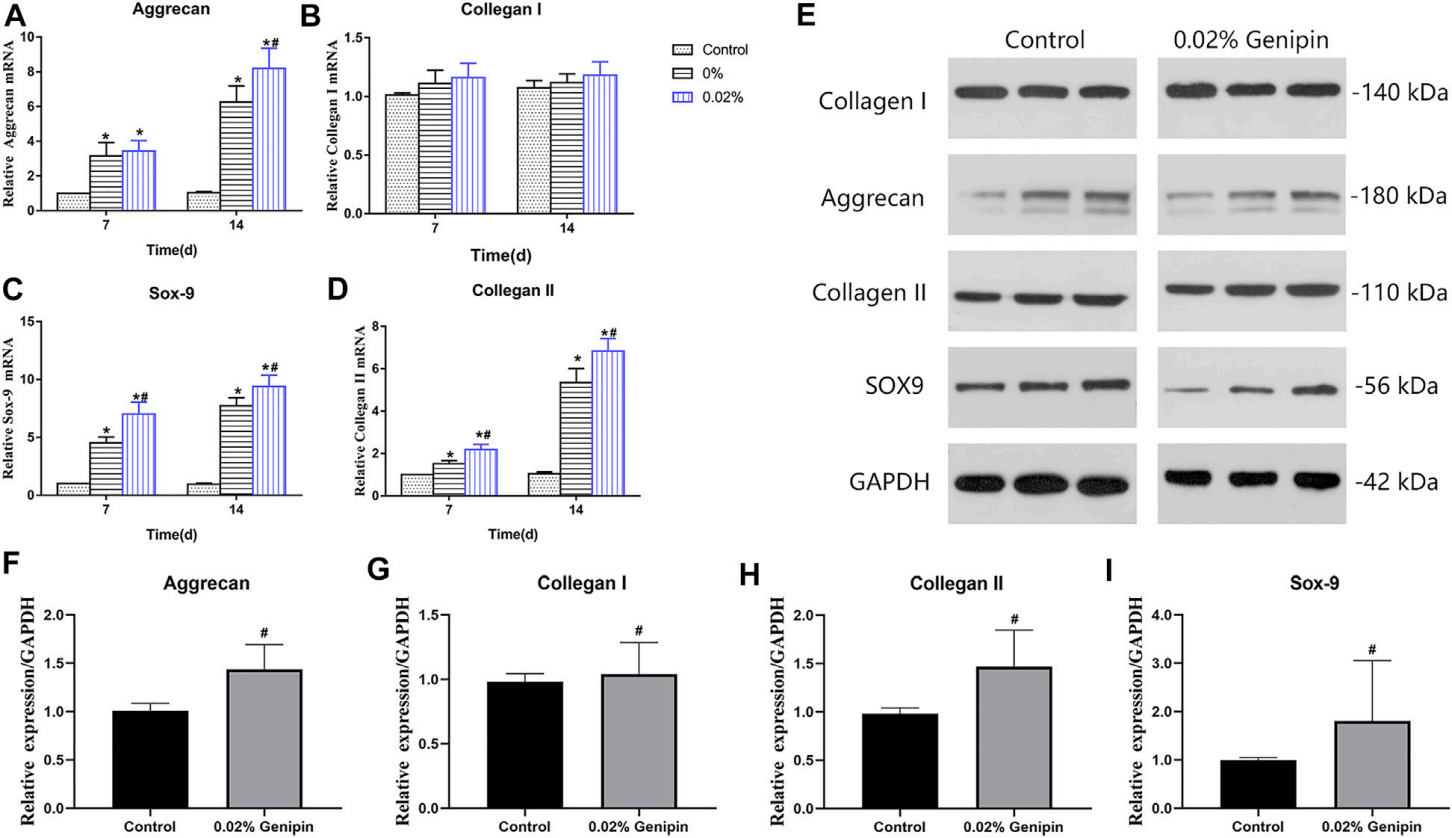
Nucleus pulposus-related gene expression levels of ADSCs. Gene expression levels of Acan **(A)**, Col-I **(B)**, Sox9 **(C)**, and Col-II **(D)** were measured at 7 and 14 days. Verification of Western blot for NP-related gene expression at 7 days is shown in **(E–I)**. Data represented mean ± SD (*n* = 5); *p < 0.05, vs. 0% group; ^#^ *p* < 0.05, vs. 0.2% group.

As shown in Figure 5A–D, the expression of NP-related genes, collagen type I (Col-1), collagen type II (Col-2), aggrecan (Acan), and sox9 showed significant differences in each group (*p* < 0.05) at 7 and 14 days, while there were no significant differences in Col-1 between each group’s expression at various time-points (*p* > 0.05). The comparison of aggrecan expression between the 0% and 0.02% groups showed no statistical difference (*p* > 0.05) at 7 days but increased significantly at 14 days in the 0.02% group (*p* < 0.05). The Col 2 and sox-9 expression levels in the 0.02% group were higher than those of other groups at each time. The expression of NP-related phenotype genes in ADSCs was highest in group 0.02%, followed by the 0% group, and lowest in the control group. In addition, the distinct effects of GDH with different concentrations of genipin in inducing differentiation of ADSCs to NP-like cells were confirmed by Western blot (Figures 5E–I).

### X-Ray

**FIGURE 6:**
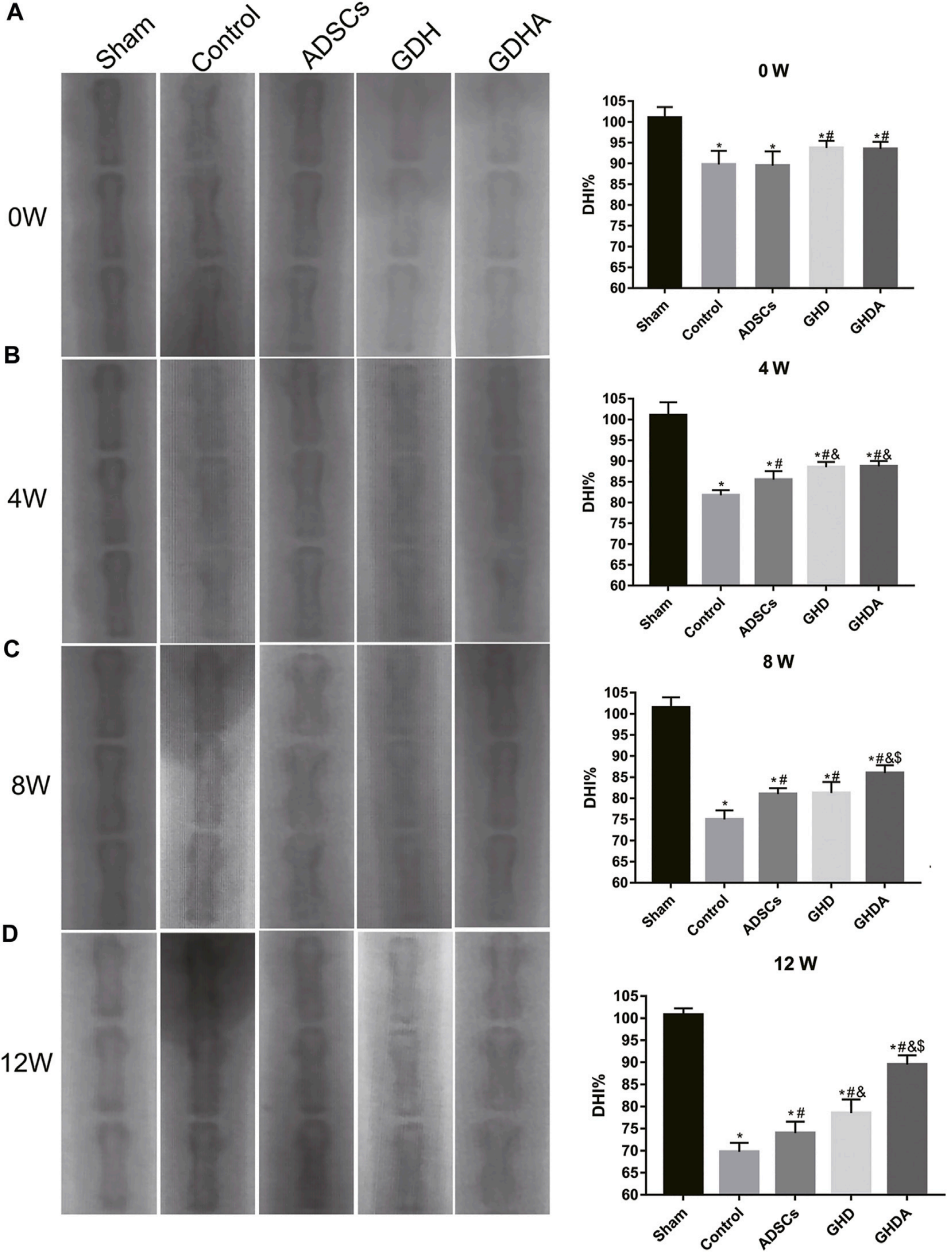
Radiographs of the caudal vertebra and change of the DHI tested by X-ray at 0 **(A)**, 4 **(B)**, 8 **(C),** and 12 **(D)** weeks after injection. Data represented mean ± SD (*n* = 5); *p < 0.05, vs. sham group; ^#^ *p* < 0.05, vs. control group; ^&^ *p* < 0.05, vs. ADSCs group; ^$^ *p* < 0.05, vs. GHD group.

The changes in X-ray and DHI in each group are shown in Figure 6. DHIs in the control group, ADSC group, GDH group, and GDHA group were significantly lower than that in the sham group at week 0 (*p* < 0.05). Compared with the control group, ADSC group, and GDH group, the DHI in the GDHA group was significantly higher at 4, 8, and 12 weeks (*p* < 0.05). Compared with the control group, DHIs in GDH group and GDHA group were much higher at 0, 4, 8, and 12 weeks (*p* < 0.05).

### MRI

**FIGURE 7:**
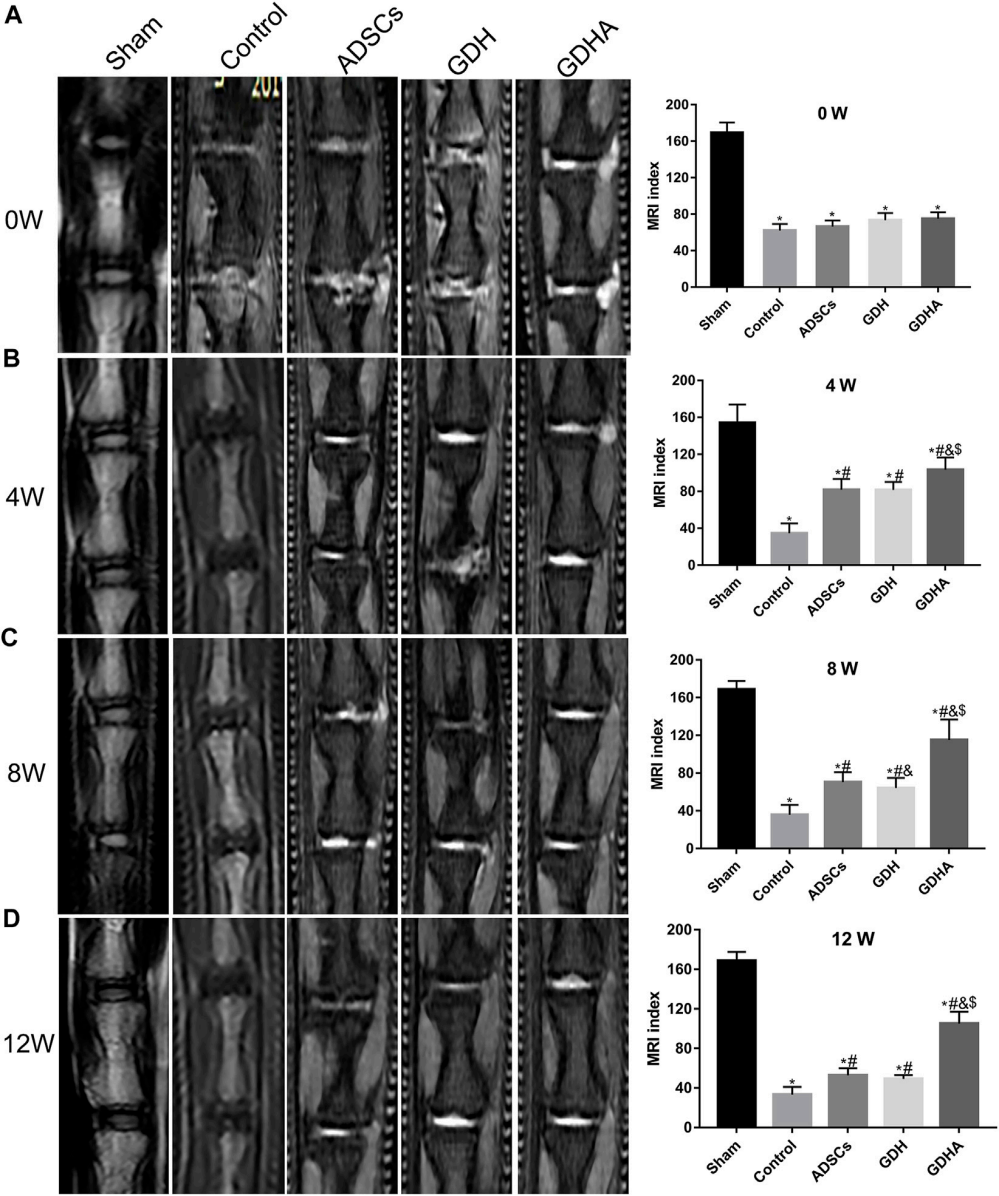
Typical MRI and MRI index change in the tail discs at 0 **(A)**, 4 **(B)**, 8 **(C),** and 12 **(D)** weeks after injection. Data represented mean ± SD (n = 5); *p < 0.05, vs. sham group; ^#^ *p* < 0.05, vs. control group; ^&^ *p* < 0.05, vs. ADSCs group; ^$^ *p* < 0.05, vs. GHD group.

The MRI reflects the structure and water content of the IVD (Figure 7). In the sham group, the structure of the IVD was intact, while in other groups, the disc was damaged to varying degrees accompanied by the T2 signal change of the NP. The MRI indexes were compared and sorted. Compared with the sham group, the MRI indices of the control group, ADSC group, GDH group, and GDHA group were significantly lower at 0, 4, 8, and 12 weeks (*p* < 0.05), respectively. The structure and T2 signal of IVD in the ADSC group, GDH group, and GDHA group were improved compared with the control group (*p* < 0.05), while the MRI indices of the GDHA group at 4, 8, and 12 weeks were much higher than those in the control group, ADSC group, and GDH group (*p* < 0.05).

### Histological Staining and Grading

**FIGURE 8:**
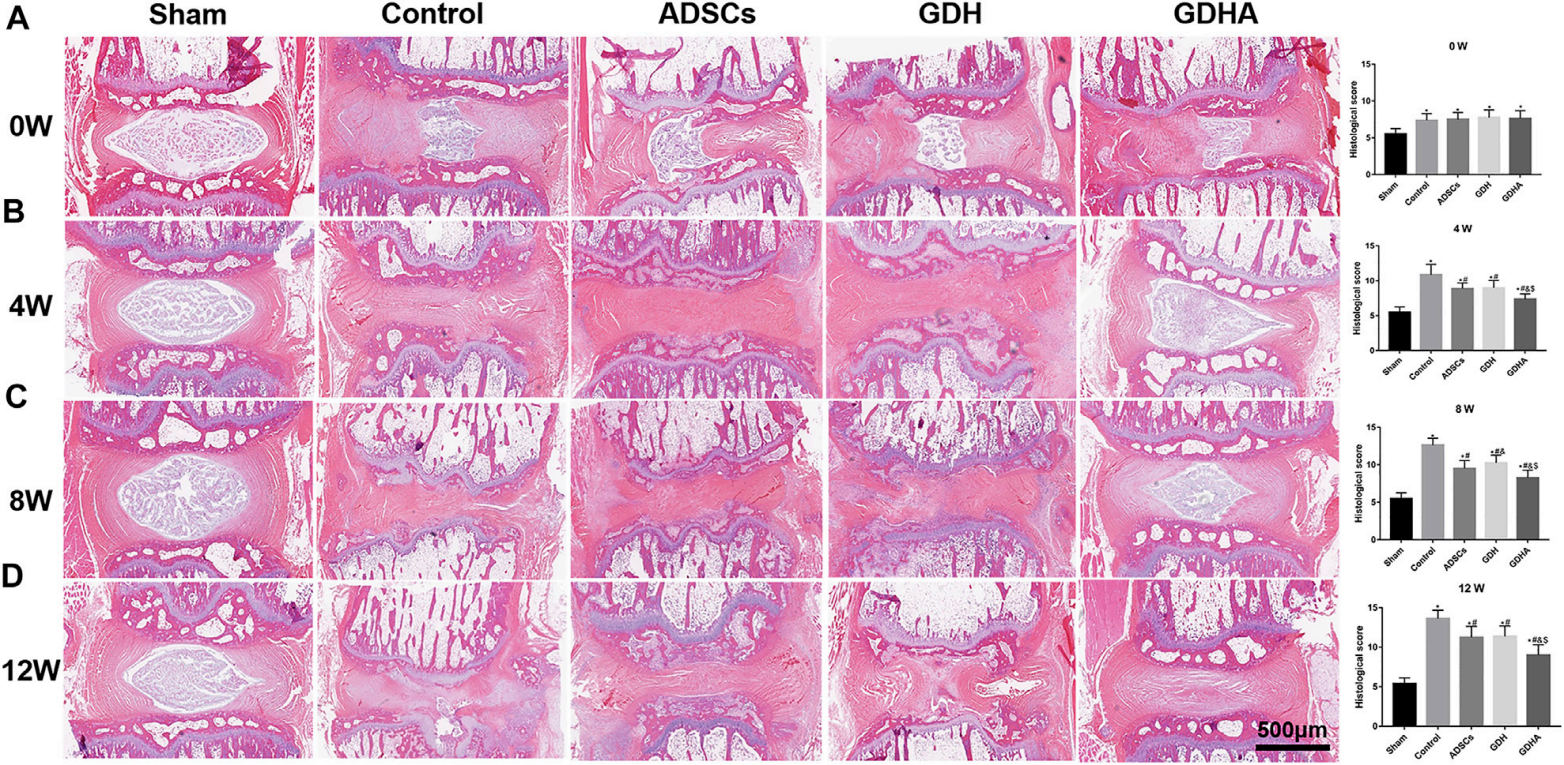
Typical HE staining and histologic score change in the tail discs at 0 **(A)**, 4 **(B)**, 8 **(C),** and 12 **(D)** weeks after injection. Data represented mean ± SD (*n* = 5); *p < 0.05, vs. sham group; ^#^ *p* < 0.05, vs. control group; ^&^ *p* < 0.05, vs. ADSC group; ^$^ *p* < 0.05, vs. GHD group. Scale bar = 500 μm.

**FIGURE 9:**
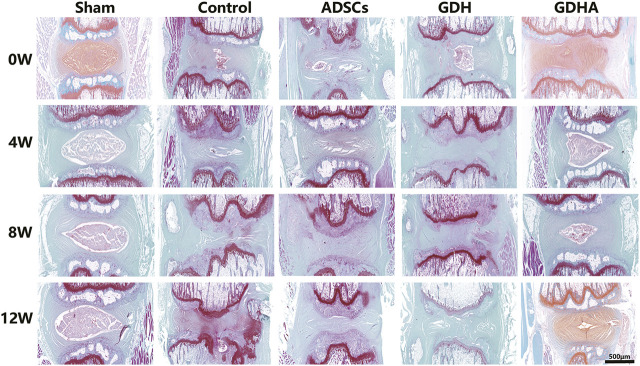
Typical Safranin O–fast green (S–O) staining in the tail discs at 0 **(A)**, 4 **(B)**, 8 **(C),** and 12 **(D)** weeks after injection. Scale bar = 500 μm.

As shown in HE staining (Figure 8) and S and O staining (Figure 9), the histological structure of IVD (distribution of NP cells, NP content and disc height) in the sham group was regular, and the corresponding histological grading score was 5. In other groups, the disordered structure of NP and annulus fibrosus was presented. The degeneration site was occupied by fibrocartilage tissue and cells with different cell morphology or even the scar tissue. The histological grading score in each group over time is shown in Figure 8B. At the advanced stage (8–12 weeks), the cell distribution and tissue structure of the GDHA group were more regular and the histological grading score was significantly improved compared with other groups (except the sham group).

### Cell Tracing

**FIGURE 10:**
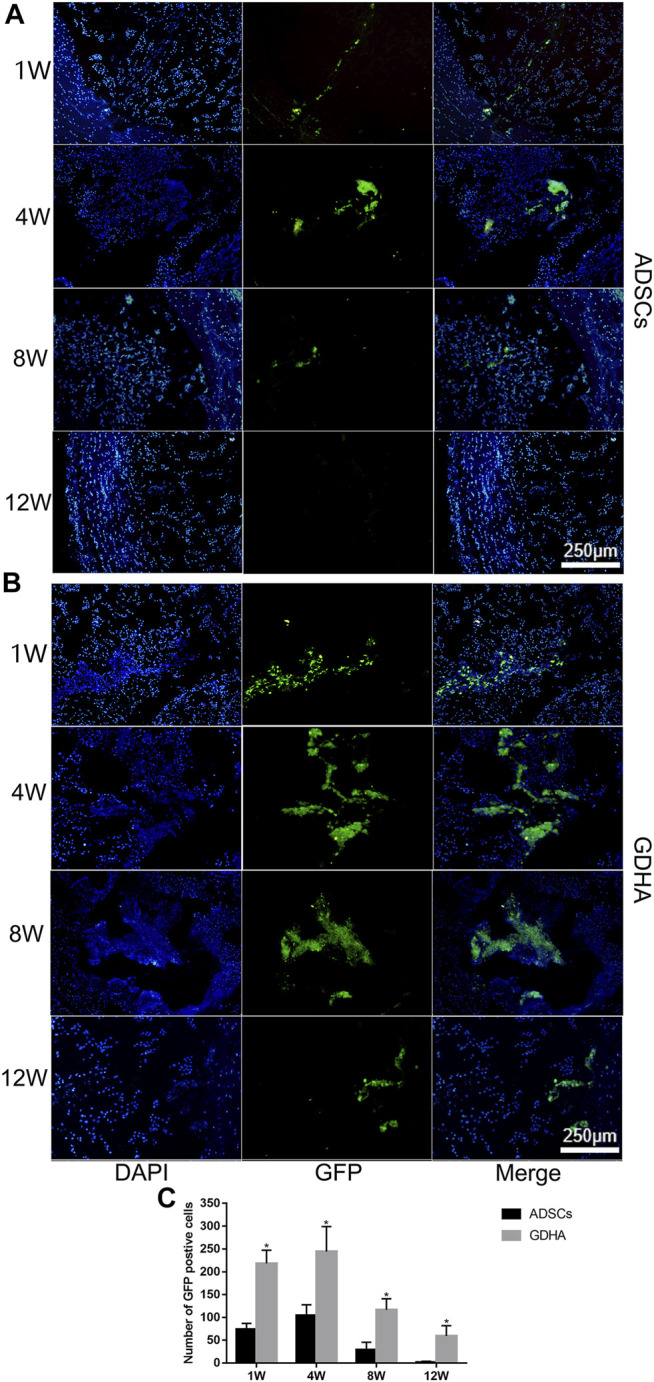
The survival and migration of ADSCs in the tail discs of the ADSC group **(A)** and GDHA group **(B)** at 1, 4, 8, and 12 weeks after injection. Data represented mean ± SD (*n* = 5); *p < 0.05, vs. sham group; ^#^ *p* < 0.05, vs. control group; ^&^ *p* < 0.05, vs. ADSC group; ^$^ *p* < 0.05, vs. GHD group. Scale bar = 250 μm.

For detecting the living ADSCs in GDH, we applied GFP-tagged ADSCs in building GDHA. As shown in Figure 10A, a small number of GFP-ADSCs with limited migration were observed, in the single ADSC group at 1 and 4 weeks. The number of cells reached the maximum at 4 weeks while it significantly decreased at 8 weeks and almost vanished at week 12 (*p* < 0.05, Figure 10C). In the GDHA group, GFP-ADSCs could survive in the degenerative disc and migrate to the surrounding area at weeks 1, 4, 8, and 12 (Figure 10B). The number of GFP-positive cells was highest at 1 and 4 weeks while it decreased gradually at 8 and 12 weeks (*p* < 0.05, Figure 10C).

## Discussion

The NP-derived ECM hydrogels offered the possibility of regeneration to degenerate NP (Collin et al., 2011; Wang et al., 2014). However, the low elastic modulus and poor stability of the ECM hydrogels may suffer no stress and cannot sustain cells for long-term survival in degenerated IVD (Chung and Burdick, 2008). Wachs et al. (2017) and Mercuri et al. (2013) reported the study of NP ECM hydrogels *in vitro* but neglected the critical mechanical properties in IVDs (Setton and Chen, 2004). It is of great significance to improve the stability and mechanical properties of the ECM hydrogel and to clarify its effects on cell behavior and relative mechanisms. In the present study, we constructed ECM-based DNPH scaffold and made it be cross-linked with genipin to improve the mechanical mechanics and stability. Afterward, we figured out a suitable genipin cross-linking concentration (0.02%) and developed a novel genipin cross-linked decellularized NP hydrogel-like cell delivery system (DNPH) to deliver ADSCs into IVD. We demonstrated that this ADSC–genipin-DNPH complex could retard IDD significantly.

Cross-linking is a well-established technique in materials science and bioengineering, through which two or more molecules are chemically connected to enhance the mechanical strength of tissues or biomaterials. In recent years, cross-linking has been gradually applied in the research of IDD and repair and continuously developed as a novel therapeutic strategy for the restoration of NPCs and ECM. As an essential factor for chemical cross-linking, the current available agents include glutaraldehyde, glyceraldehyde, ribose, and glucose (Wollensak and Spoerl, 2004). The biological toxicity of chemical cross-linking agents cannot be ignored during the cross-linking process, which limits their further clinical application (Sung et al., 1999). As an extract from traditional Chinese medicine, Eucommia ulmoides, genipin is a natural biological cross-linking agent with low toxicity. Due to its mechanical-enhancing properties and biocompatibility of the matrix (Muzzarelli et al., 2016), genipin has been widely used in the bioengineering research of sclera, articular cartilage, tendon, and bone (Kim et al., 2010; Yan et al., 2010; Muzzarelli et al., 2015; Zhu et al., 2016). In the field of bioengineering repair for IDD, the application of genipin is still in the initial stage (Zhou et al., 2018a; Zhou et al., 2018b; Fujii et al., 2020). Zhou et al. produced the genipin cross-linked type II collagen/chondroitin sulfate composite scaffold, which had the property of *in-situ* gelation and induced adipose-derived stem cells differentiating to an NP-like phenotype *in vitro* (Zhou et al., 2018b). In addition, Zhou et al. developed a genipin-cross-linked type II collagen scaffold and found that it can promote the differentiation of adipose-derived stem cells into NP-like cells (Zhou et al., 2018a). However, whether GDH has the therapeutic effect on IDD is hitherto unclear. In the present study, the gelation kinetics and ninhydrin detection showed that the GDH was initially self-cross-linking rapidly at the first half hour, then slowed down by genipin, and finally completed in around 48 h. The cross-linking degree, elastic modulus, and resistance to enzymatic hydrolysis of the GDH were positively correlated with genipin concentration. SME showed that the mesh area in the hydrogel decreased with the increase in genipin concentration, suggesting that genipin may establish more solid cross-linking to the hydrogel. When the concentration of genipin is 0.02%–0.04%, the elastic modulus of GDH is close to human NP (about 11 kPa) (Iatridis et al., 1996).

The CCK-8 test showed that there was no significance between different groups (expect the 0.04% genipin group) at various time-points. Meanwhile, the dead/live test showed that most of the ADSCs cultured on the GDH with low concentrations of genipin (0%–0.02%) were living, which account for about 90%. However, in the 0.04% group, it showed a significant increase of dead cells, and the ratio of living cells was only 50% as well. According to these results, we deduced that the optimum concentration of genipin is 0.02%.

The gene expression of NP-related phenotypic molecules (aggrecan, Sox-9, collagen I, and collagen II) represents the matrix synthesis and reflects the NP-like differentiation of ADSCs. The RT-PCR and Western blot showed there were no significant between different groups in the expression of *Col-1* (NP-negative maker) at various time-points. Compared with the control group, gene expression levels of NP-positive markers (*Col-2*, Acan, and Sox-9) were increased in the DNPH group and reached to the highest level in the 0.02% genipin–DNPH group. This suggested that DNPH promoted the NP-like differentiating induction of DNPH which could be enhanced by genipin cross-linking. There might be several potential mechanisms contributing to this phenomenon. Firstly, ECM cross-linked by genipin leads to variations in stiffness and roughness, while its proteic composition remains unchanged (Yoo et al., 2011). Its inherent collagen II could improve the differentiation of ADSCs into NP cells (Yin et al., 2018) and promote the synthesis of ECM (Tao et al., 2016; Zhou et al., 2018b). Secondly, the stiffness of the ECM leads to the differentiation of MSCs. The mechanically related transcription coactivator (YAP/TAZ) with a PDZ-binding motif has been shown to influence the behavior of MSC by transducing signals originated from ECM mechanical cues (Dupont, 2016). Previous studies showed that MSCs have robust osteogenic capacity on a rigid matrix with a modulus of 40 kPa (Ye et al., 2015; Ye et al., 2016), while on a soft matrix (0–1 kPa) they contribute to adipogenic differentiation (Engler et al., 2006). In this study, the modulus of GDH (with 0.02% genipin) was about 11 kPa, contributing to chondroid differentiation (Akhmanova et al., 2015). Thirdly, the secondary structure and surface properties of ECM were changed in GHD (Qiu et al., 2013). SEM showed that loose mesh in DNPH changed into denser mesh in GDH. In the perspective of topological structure, surface characteristics and pore size may affect the differentiation of stem cells (Ma et al., 2018). Nevertheless, the molecular mechanism in which GDH (with 0.02% genipin) exerted specific NP differentiation–induction on ADSCs needs to be explored in further studies.

Given the differences between *in vitro* and *in vivo* environments, whether ADSCs can survive and maintain the same cytoactivity in degenerated IVD as the *in vitro* culture needs to be clarified. In the present study, the *in vivo* cell tracing showed that a few ADSCs in the ADSCs group were alive at 1 and 4 weeks, while in the GDHA group, more cells were still alive at 8 weeks and kept cytoactive, indicating that GDH could provide vital support for surviving ADSCs in IVD. Moreover, our results also showed that GDHA significantly restored the histological characteristics of degenerated IVD. Compared with the degenerative group, the intervertebral height and structure in the ADSC group were improved, while they were still worse than those in the GDHA group. Some studies have shown that the degraded NPs possess a detrimental environment (low oxygen tension, limited nutrition, acidic pH, high osmotic pressure), which has a negative impact on the function and viability of transplanted cells (Henry et al., 2018). As abovementioned, only a few ADSCs survived at 4 weeks. Considering that related ECM can be synthesized by ADSCs in the early stage, it is difficult to make ADSCs survive in the long term in harsh environments and hardly achieve continuous restoration of the IVD physiological structure. The cell tracer showed that the cells in the GDHA group could survive longer, combined with a higher elastic modulus. The DHI, MRI index, and degeneration index were improved in the GDHA group, but they were still inferior to those in the sham group. In addition, due to the improved elastic modulus and better anti-enzymatic hydrolysis performance, the intervertebral height and T2 signal values in the GDH group were higher than those in the degenerative group. Therefore, GDHA possesses superior mechanical properties and can prolong the survival time of ADSCs, which is an ideal biomaterial for IVD regeneration.

## Conclusion

In the present research, we developed a novel genipin cross-linked decellularized NP hydrogel-like cell delivery system to deliver ADSCs into IVD. The GDH is cross-linked with 0.02% genipin and has the ability of *in situ* gelation after injection. The GDHA cross-linking degree is moderate with an increased bio-stability and solid-like properties after gelation. Our *in vitro* results demonstrated the bio-safety of the GDHA and the stimulating effects of the GDHA on the differentiation of ADSCs to NP-like cells. We further studied the pro-regenerative effects of the GDHA delivery system on the IDD in the rat model, and we demonstrated that it could partly promote the regeneration of the degenerated NP. We hope our study can provide new clinical application of stem cell-based tissue engineering for the treatment of IDD.

## Data Availability

The raw data supporting the conclusions of this article will be made available by the authors, without undue reservation.
