# Supplementary material for: Genipin Cross-Linked Decellularized Nucleus Pulposus Hydrogel-Like Cell Delivery System Induces Differentiation of ADSCs and Retards Intervertebral Disc Degeneration
Source: Front Bioeng Biotechnol. 2021 Dec 23;9:807883. doi: 10.3389/fbioe.2021.807883 (PMC8733700; doi:10.3389/fbioe.2021.807883)
Supplement: Supplementary file 1 [file DataSheet1.pdf]

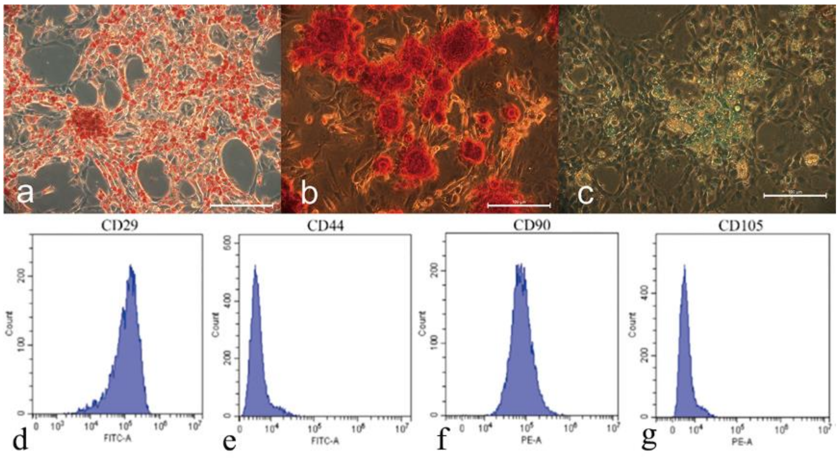

Supplement fig.1 Differentiation and flow cytometry experiments. ADSCs were identified by differentiation (a, b, c) and flow cytometry (d, e, f, g). a: oil red O staining for adipocytes differentiation; b: alizarin red staining for osteoblast differentiation; c: alcian blue staining for chondrocytes differentiation. The expression rate of CD29(d) and CD90(f) >90%, the expression rate of CD44(f) and CD45(g) <5%. Scale bar = 100µm.
